# Hydrophobia

**Published:** 1888-03

**Authors:** 


HYDROPHOBIA is getting uncomfortably frequent in many sections of our country. The opinion is now advanced that the bite of a dog not rabid is unsafe when there is a wide spread prevalence of the disease; their being occasional cases reported of the disease having developed from a supposed healthy dog. There may be something in this idea as it is conceded that in certain edpidemics, as of cholera, influenza, etc., there is always found a predisposition to the epidemic or a modified form even among those not directly exposed to or in immediate contact with the prevailing malady. Due, it is supposed, to what Sydenham calls "The Epidemic Constitution of the Atmosphere."

